# Umbilical Hernia Treated with Adhesive Bands

**Published:** 1846-02

**Authors:** 


*Umbilical Hernia treated with adhesive bands.*—A recent No. of the "*Journal de Medicine de Bruxelles*" contains the following details of Dr. Seutin's method of treating umbilical hernia in children: "The hernia having been reduced, the surgeon places a small pad over the orifice and retains it with his index finger, whilst with his thumb and middle finger he draws up a fold of skin on each side of it longitudinally. The band of adhesive plaster is then applied, one end being applied by an assistant to the lumbar region, and held there until the surgeon can carry the other end over the cutaneous folds and pad, and finally to the opposite lumbar region. A roller bandage is then passed several times around the abdomen in order to secure the adhesion of the plaster, and may be removed in a few hours. Mr. S. renews this bandage every two or three weeks, and if an eruption is produced, one end of the plaster may be raised so as to allow the application of a little cerate, without removing the whole."
--*Ibid.*

Professor Dugas informs us that he has been in the habit of treating umbilical hernia with adhesive plaster for the last six years, with uniform success. Prof. D. does not, however, proceed as recommended by Dr. Seutin, but simply carries a band of adhesive plaster, three inches wide and from eight to twelve inches long, across the abdomen, by first placing the center of the plaster over the reduced hernia, and maintaining it there with one hand, whilst with the other he extends the ends to each side of the body, carefully avoiding the formation of any cutaneous folds. In some cases another plaster of similar dimensions is laid over the first, in order to increase the stiffness of the application. In but one or two cases has Prof. D. resorted to a small compress of old linen over the umbilicus. According to Prof. D., the plasters are to be renewed whenever they cease to adhere firmly, or occasion an eruption--in which latter case, the plaster may be applied obliquely so as not to cover the same surface, save that about the umbilicus, which may then be covered with a bit of linen and simple cerate for a few days.

By thus applying to the skin a firm and unyielding covering, its own elasticity is overcome, and the protrusion consequently prevented.—*Ibid.*

